# Association of Long-term Outcomes and Survival With Multidisciplinary Salvage Treatment for Local and Regional Recurrence After Stereotactic Ablative Radiotherapy for Early-Stage Lung Cancer

**DOI:** 10.1001/jamanetworkopen.2018.1390

**Published:** 2018-08-24

**Authors:** Eric D. Brooks, Bing Sun, Lei Feng, Vivek Verma, Lina Zhao, Daniel R. Gomez, Zhongxing Liao, Melenda Jeter, Michael O’Reilly, James W. Welsh, Quynh-Nhu Nguyen, Jeremy J. Erasmus, George Eapen, Kamran Ahrar, Mara B. Antonoff, Stephen M. Hahn, John V. Heymach, David C. Rice, Joe Y. Chang

**Affiliations:** 1 Department of Radiation Oncology, The University of Texas MD Anderson Cancer Center, Houston; 2 Department of Biostatistics, The University of Texas MD Anderson Cancer Center, Houston; 3 Department of Radiation Oncology, University of Nebraska Medical Center, Omaha; 4 Department of Diagnostic Radiology, The University of Texas MD Anderson Cancer Center, Houston; 5 Department of Pulmonary Medicine, The University of Texas MD Anderson Cancer Center, Houston; 6 Department of Interventional Radiology, The University of Texas MD Anderson Cancer Center, Houston; 7 Department of Thoracic and Cardiovascular Surgery, The University of Texas MD Anderson Cancer Center, Houston; 8 Department of Thoracic Head and Neck Medical Oncology, The University of Texas MD Anderson Cancer Center, Houston

## Abstract

**Importance:**

Stereotactic ablative radiotherapy (SABR) is first-line treatment for patients with early-stage non–small cell lung cancer (NSCLC) who cannot undergo surgery. However, up to 1 in 6 such patients will develop isolated local recurrence (iLR) or isolated regional recurrence (iRR). Little is known about outcomes when disease recurs after SABR, or about optimal management strategies for such recurrences.

**Objective:**

To characterize long-term outcomes for patients with iLR or iRR after SABR for early-stage NSCLC with the aim of informing treatment decision making for these patients with potentially curable disease.

**Design, Setting, and Participants:**

In this cohort study, a retrospective review was conducted of 912 patients prospectively enrolled in an institutional database at a tertiary cancer center from January 1, 2004, through December 31, 2014.

**Main Outcomes and Measures:**

Overall survival, progression-free survival, recurrence patterns, demographics, salvage techniques, patterns of salvage failure, and toxic effects.

**Results:**

Of the 912 patients in the study (456 women and 456 men; median age, 72 years [range, 46-91 years]), 756 (82.9%) had T1 tumors at initial diagnosis; 502 tumors (55.0%) were adenocarcinomas and 309 tumors (33.9%) were squamous cell carcinomas. Of 912 patients with early-stage I to II NSCLC who received definitive SABR (50 Gy in 4 fractions or 70 Gy in 10 fractions), 102 developed isolated recurrence (49 with iLR and 53 with iRR), and 658 had no recurrence. Median times to recurrence after SABR were 14.5 months (range, 1.5-60.8 months) for iLR and 9.0 months (range, 1.9-70.7 months) for iRR; 39 of 49 patients (79.6%) with iLR and 48 of 53 patients (90.6%) with iRR underwent salvage with reirradiation, surgery, thermal ablation, or chemotherapy. Median follow-up times for patients with iLR or iRR were 57.2 months (interquartile range, 37.7-87.6 months) from initial SABR and 38.5 months (interquartile range, 19.9-69.3 months) from recurrence. Rates of overall survival at 5 years from initial SABR were no different between patients with iLR and salvage treatment (57.9%) and patients with no recurrence (54.9%; hazard ratio, 0.89; 95% CI, 0.56-1.43; *P* = .65) but were lower for patients with iRR and salvage treatment (31.1%; hazard ratio, 1.43; 95% CI, 1.00-2.34; *P* = .049). Patients receiving salvage treatment had longer overall survival than patients who did not (median, 37 vs 7 months after recurrence; hazard ratio, 0.40; 95% CI, 0.09-0.66; *P* = .006). Twenty-four of 87 patients (27.6%) who received salvage treatment for iLR or iRR subsequently developed distant metastases. No patient experienced grade 5 toxic effects after salvage treatment.

**Conclusions and Relevance:**

Life expectancy after salvage treatment for iLR was similar to that for patients without recurrence, but survival after salvage treatment for iRR was similar to that of patients with stage III NSCLC. Patients who received salvage treatment had significantly improved survival. Because salvage treatment for iLR or iRR was based on a consistent multidisciplinary approach, this may help in clinical decision making.

## Key Points

**Question:**

What are the outcomes for potentially curable local or regional recurrences after stereotactic ablative radiotherapy for early-stage non–small cell lung cancer?

**Findings:**

In this cohort study of 912 patients who received stereotactic ablative radiotherapy for early-stage I to II non–small cell lung cancer, salvage treatment was significantly associated with improved survival. Survival for patients with local recurrence who received salvage treatment was no different than that for patients with no recurrence, and survival for patients with regionally recurrent disease was similar to that for patients with stage III disease.

**Meaning:**

This study provides data on outcomes for patients with recurrence after stereotactic ablative radiotherapy, which may help in clinical decision making.

## Teaser

This cohort study examines long-term outcomes for patients with isolated local recurrence or isolated regional recurrence after stereotactic ablative radiotherapy for early-stage non–small cell lung cancer with the aim of informing treatment decision making.

## Introduction

Historically, stereotactic ablative radiotherapy (SABR)—giving small numbers of high ablative doses of radiotherapy over a short period—has produced long-term rates of control of local and regional disease exceeding 80% when it is used as first-line treatment for appropriately chosen patients with early-stage non–small cell lung cancer (NCSLC).^1^ Stereotactic ablative radiotherapy has recently been shown to produce survival and cancer-specific outcomes comparable with those of patients who have undergone lobectomy, but with less morbidity, and today represents first-line therapy for patients whose disease is inoperable.^2,3,4^ Although the use of SABR for patients with operable disease remains under investigation, the elderly population is composing a greater proportion of all patients treated; as such, the number of patients with early-stage NSCLC that is inoperable, and thus deferred to definitive SABR treatment, continues to rise.^5,6^

Recurrence patterns after SABR have been reported, but, to date, outcomes have not.^4^ Until now, little was known about the 1 in 6 patients who develop isolated local recurrence (iLR) or isolated regional recurrence (iRR) after first-line SABR.^1,2,3,4^ Thus, for thoracic oncologists, clinical questions about the outcomes for such patients (whose disease is potentially curable) and how best to manage recurrences have remained largely unanswered.

Although options for treating recurrence (such as surgery and reirradiation) are offered in guidelines from the National Comprehensive Cancer Network^7^ and the European Society for Medical Oncology,^8^ they tend to not apply easily to the population of patients undergoing SABR, most of whom were not candidates for surgery and had already received radiotherapy. Thus, no evidence-based guidelines or large-scale studies specifying how to determine when a given salvage technique would be appropriate for these patients have been available. Moreover, since much of the evidence to support salvage treatment after SABR has been limited to studies of small, heterogeneous groups of patients,^9,10,11,12,13,14,15,16,17,18,19,20,21,22,23^ little is known of outcomes after iLR or iRR after SABR for early-stage NSCLC.

We sought to fill that void by reporting long-term outcomes for a large group of patients with iLR or iRR after SABR for early-stage NSCLC. Our findings on survival, disease control, and toxic effects after various salvage techniques serve to inform treatment decision making for these patients with potentially curable disease.

## Methods

### Patients

We analyzed 912 patients with clinical stage T1 to T3 (satellite nodule) N0M0 NSCLC not involving the bronchial tree or other critical structures, who had received image-guided SABR on an institutional protocol at MD Anderson Cancer Center, Houston, Texas, from January 1, 2004, through December 31, 2014. All patients had been registered prospectively, and their records were reviewed retrospectively for this analysis, which took place from June 1 to August 30, 2017. Before SABR, disease was staged by chest computed tomography (CT) and positron emission tomography (PET) with CT, with brain CT or magnetic resonance imaging as needed. Images suggesting mediastinal disease were followed up with endobronchial ultrasound–guided biopsy to rule out nodal metastases. This study was approved by the MD Anderson Cancer Center institutional review board, and the requirement for informed consent was waived owing to deidentification of patient data. This study followed the Strengthening the Reporting of Observational Studies in Epidemiology (STROBE) reporting guidelines.^24^

### SABR Protocol

Four-dimensional CT images were obtained in all cases to account for tumor motion, and respiratory gating was used for patients whose tumor moved more than 1 cm. Most patients were treated with 50 Gy in 4 fractions (to convert gray to rad, multiply by 100), except for patients with large or central lesions (ie, within 2 cm of critical mediastinal structures or the brachial plexus), for whom dose-volume constraints for normal tissues could not be achieved. Such patients were treated with 70 Gy in 10 fractions or other regimens with a lower biologically effective dose.^25,26^ Doses (eg, 50 Gy in 4 fractions or 70 Gy in 10 fractions) were typically prescribed to the 70% to 90% isodose line covering the planning treatment volume (PTV).

For plans for intensity-modulated radiotherapy or volumetric modulated arc therapy, an integrated boost to the gross tumor volume brought the total dose to 60 Gy in 4 fractions or 85 Gy in 10 fractions; this boost was done to mimic 3-dimensional conformal radiation–based SABR planning to generate a high-dose region inside the gross tumor volume. Treatment was delivered on consecutive weekdays with a break on intervening weekend days, if applicable. Other SABR treatment planning and delivery details have been previously described.^27^

### Follow-up Evaluations and Definitions of Treatment Failure

Follow-up evaluations after initial SABR included chest CT scans every 3 months for the first 2 years, every 6 months for the next 3 years, and annually thereafter. Scanning with PET and CT was commonly performed at 3 to 12 months after SABR to evaluate response and detect early recurrence. Local recurrence (LR) was defined as evidence on CT of progressive soft-tissue abnormalities in the same lobe as the primary tumor that then corresponded to areas avid on PET or positive biopsy findings.^28^ Regional recurrence (RR) was defined as similar CT, PET, or biopsy findings in the hila or mediastinum. Recurrence in previously uninvolved lobes or outside the thorax was defined as distant failure. Isolated local recurrence and iRR were defined as LR or RR with no other recurrence. In-field LRs were within 1 cm of the initial SABR PTV, marginal LRs overlapped with the PTV plus 1 cm, and out-of-field LRs appeared beyond the PTV plus 1 cm. Any patient with confirmed LR or RR also received PET, brain magnetic resonance imaging, and/or mediastinal endobronchial ultrasonography as indicated for restaging.

Second primary lung carcinomas were defined by the modified Martini and Melamed criteria^29^ as a new tumor of different histologic or molecular subtype, or a new tumor of the same histologic characteristics in a different lobe appearing after a tumor-free interval of more than 2 years.^3^ All cases were reviewed before initial SABR and at recurrence by a multidisciplinary treatment team (D.R.G., Z.L., M.J., M.O., J.W.W., Q.-N. N., J.J.E., G.E., K.A., M.B.A., S.M.H., J.V.H., D.C.R., and J.Y.C.) consisting of thoracic surgeons, medical oncologists, radiation oncologists, interventional radiologists, pulmonologists, and radiologists. All available information was reviewed, including pathologic findings, clinical history, and imaging features. In all cases, this team determined which treatments were possible and reached consensus on a preferred treatment approach for each patient, as described below.

### Salvage Therapy

**Figure 1.**
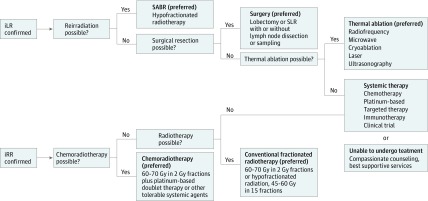
Management Guide for Treating Isolated Local Recurrence (iLR) and Isolated Regional Recurrence (iRR) After Stereotactic Ablative Radiotherapy (SABR) for Early-Stage Non–Small Cell Lung Cancer The workup involves positron emission tomography with computed tomography, magnetic resonance imaging of the brain, endobronchial ultrasound (if applicable based on findings of imaging), liver function tests, complete blood cell count, basic metabolic panel, and pulmonary function tests (if surgery is considered). Systemic therapy may be carefully considered in conjunction with locally directed therapy for iLR or iRR given the rates of distant metastases observed. SLR indicates sublobar resection.

In all cases, the choice of salvage therapy for iLR or iRR was made via consistent multidisciplinary evaluation. The process for salvage therapy selection and management approach is summarized in Figure 1.

For iLR, repeated SABR was the preferred salvage therapy because of its low morbidity.^23^ Repeated SABR was possible when the iLR was sufficiently far from critical central chest structures^25^ or was outside the original SABR treatment volume (>1 cm from the initial SABR PTV). For iLRs encroaching on prior treatment fields (ie, marginal recurrences), an alternative to 50 Gy in 4 fractions (often 70 Gy in 10 fractions) was used for safety. All marginal recurrences for repeated SABR were peripheral and away from central chest structures and were discussed in a multidisciplinary setting to determine if the cumulative dose to the prior irradiated volume was safe. For patients who were not candidates for repeated SABR, or who were candidates for surgery, surgical resection was the next preferred option.^19,20,21,22^ All patients considering surgery had sufficient pulmonary function (predicted postoperative diffusing capacity for carbon monoxide and forced expiratory volume in 1 second >40%) and were deemed adequate risk candidates by a thoracic surgeon. For iLRs that could not be safely treated with reirradiation or with surgery, thermal ablation was preferred. Currently, thermal ablation can be done percutaneously, is suitable for lesions up to 3 cm in diameter, and can be used on tumors 1 cm or more from central chest structures.^30,31,32,33^ Patients undergoing thermal ablation must be able to safely tolerate a small pneumothorax.

For patients with iRR, bimodality treatment with nodal irradiation and systemic therapy was preferred. This approach is similar to that for patients with stage II or III NSCLC presenting with node-positive disease. The most common systemic therapy was platinum-paclitaxel doublet therapy, and local control was attempted with conventional radiotherapy to the involved nodes. Doses of 60 to 70 Gy in 2-Gy fractions were preferred, but in some circumstances, the dose was reduced to meet normal tissue (or patient) tolerance. Patients at high risk of complications from platinum-based doublet therapy were given mono-agent cytotoxic therapy. Patients who could not tolerate chemoradiotherapy were given either systemic therapy or radiotherapy alone. For patients unable to tolerate radiotherapy, the systemic agent was chosen based on toxic effects and appropriateness given the tumor’s molecular characteristics, and used until progression, eradication of disease, or death. If systemic therapy was not possible, definitive radiotherapy was given to a dose as close as possible to that used for stage III disease (60-70 Gy in 2-Gy fractions). Patients unable to undergo additional local or systemic therapy were given best supportive care.^34^

### Statistical Analysis

The Kaplan-Meier method was used to estimate probabilities of overall survival (OS) and progression-free survival. Overall survival was calculated from completion of SABR to death from any cause and was also calculated from time of iLR or iRR to death from any cause. Time-varying covariate analysis using recurrence as the covariate was also used when examining OS between patients with iLR or iRR and no recurrence to account for survival bias. Progression-free survival was calculated from completion of SABR to the first failure at any site or death. Times to LR, RR, or distant recurrence were calculated from completion of SABR to the development of local, regional, or distant failure as both first events and cumulatively as concurrent or subsequent events over the course of the study.

In addition to reporting crude recurrence rates, we also calculated the incidence of local, regional, and distant failure by using the Kaplan-Meier method with death as a competing risk.^35^ These criteria were also applied to reporting rates of second primary lung cancer. Treatment-related toxic effects were scored with the Common Terminology Criteria for Adverse Events, version 4.0.^36^ *P* < .05 (2-sided) was considered statistically significant. χ^2^ Analysis was used for categorical variables. Data were analyzed with SPSS, version 21.0 (IBM Corp), with a macro to calculate the cumulative incidence with competing risk.

## Results

The study population comprised 912 patients consecutively treated with SABR in 2004-2015 (Table 1 and eFigure 1 in the Supplement). The median patient age was 72 years (range, 46-91 years), 456 (50.0%) were men and 456 (50.0%) were women, 756 patients (82.9%) had clinical T1 disease, and 156 patients (17.1%) had clinical T2 or T3 disease (per the American Joint Committee on Cancer, 7th edition, guidelines).^37^ Among the 912 patients, 502 tumors (55.0%) were adenocarcinomas and 309 (33.9%) were squamous cell carcinomas. Nearly all cases (903 [99.0%]) had been confirmed by biopsy. The median follow-up time was 59.3 months (interquartile range [IQR], 37.7-87.9 months) from the initial SABR. About one-third of patients (318 [34.9%]) had staging mediastinal endobronchial ultrasonography for suspected lymphadenopathy on PET or CT (eg, node ≥1 cm). Most patients (773 [84.8%]) had been referred for initial SABR for inoperable disease or medical contraindications, and the other 139 patients (15.2%) had declined surgery or were randomized to SABR on the STARS (Stereotactic Ablative Radiotherapy [SABR] in Stage I Non-small Cell Lung Cancer Patients) protocol (ClinicalTrials.gov identifier NCT02357992). Most patients (754 [82.7%]) had good performance status, with Eastern Cooperative Oncology Group scores of 0 to 1 at diagnosis.

**Table 1.** Characteristics and Outcomes for All Patients Initially Treated With Stereotactic Ablative Radiotherapy for Early-Stage Non–Small Cell Lung Cancer

Characteristic	Patients, No. (%) (N = 912)
Age, median (range), y	72 (46-91)
Sex	
Male	456 (50.0)
Female	456 (50.0)
Tumor status	
T1	756 (82.9)
T2 (T2a: ≤5 cm, pleural invasion)	140 (15.4)
T3 (with satellite nodule)	16 (1.8)
Tumor histologic findings	
Adenocarcinoma	502 (55.0)
Squamous cell carcinoma	309 (33.9)
Other	23 (2.5)
NSC NOS	69 (7.6)
No pathologic findings obtained	9 (1.0)
Tumor location	
Peripheral	760 (83.3)
Central	152 (16.7)
SABR dose/fraction (BED)	
50 Gy/4 fractions (112.5 Gy)	720 (78.9)
70 Gy/10 fractions (119 Gy)	124 (13.6)
Others (75-180 Gy)	68 (7.5)
Endobronchial ultrasonography	
Yes	318 (34.9)
No	594 (65.1)
Reason for SABR	
Inoperable disease	773 (84.8)
Declined surgery	139 (15.2)
ECOG score	
0	87 (9.5)
1	667 (73.1)
2	146 (16.0)
3	12 (1.3)
First site of recurrence	
Isolated LR	49 (5.4)
Isolated RR	46 (5.0)
Isolated DM	96 (10.5)
Concurrent LR and RR	7 (0.8)
Concurrent LR and DM	19 (2.1)
Concurrent RR and DM	29 (3.2)
All sites failure	8 (0.9)
Cumulative recurrence of events over the entire time course	
LR	91 (10.0)
RR	105 (11.5)
DM	183 (20.1)
Time to any recurrence as first event, median (range), mo	
LR	14.9 (1.5-91.9)
RR	10.5 (1.4-70.7)
DM	11.6 (0.2-91.9)
Follow-up time, median (IQR), mo	59.3 (37.7-87.9)
Overall survival time, median (95% CI), mo	56.3 (51.4-61.2)
1-y rate, %	88.8
3-y rate, %	64.9
5-y rate, %	47.7
Progression-free survival time, median (95% CI), mo	39.7 (34.6-44.8)
1-y rate, %	78.1
3-y rate, %	52.7
5-y rate, %	39.1
Second primary lung cancer	68 (7.5)
Time to second primary lung cancer, median (range), mo	23.6 (1.2-122.4)

Abbreviations: BED, biological effective dose; DM, distant metastasis; ECOG, Eastern Cooperative Oncology Group; IQR, interquartile range; LR local recurrence; NSC NOS, non–small cell not otherwise specified; PFS, progression-free survival; RR, regional recurrence; SABR, stereotactic ablative radiotherapy.

SI conversion factor: To convert gray to rad, multiply by 100.

### Recurrence Patterns and Survival After Initial SABR

Recurrences as cumulative and first events for the 912 patients are presented in Table 1 and eFigure 2 in the Supplement. Most patients (658 [72.1%]) did not experience recurrence. First failures were iLR in 49 patients (5.4%), iRR in 46 (5.0%), and simultaneous iLR and iRR in 7 (0.8%). (For the purposes of this analysis, these 7 patients with simultaneous iLR and iRR were considered to have iRR, bringing the total number of patients with iLR or iRR to 102 [11.2%].) Distant failure as a first event, alone or in combination with other failure, was the predominant pattern of failure (152 patients [16.7%]). The median time to iLR was 14.9 months (IQR, 1.5-91.9 months), to iRR was 10.5 months (IQR, 1.4-70.7 months), and to distant failure was 11.6 months (IQR, 0.2-91.9 months). The cumulative rates of recurrence (calculated not with the Kaplan-Meier method, but rather considering subsequent events in addition to first events) were 10.0% for LR (91 of 912), 11.5% for RR (105 of 912), and 20.1% for distant failure (183 of 912). The cumulative crude rate of second primary lung cancer was 7.5% (68 of 912).

The cumulative incidence of LR, RR, distant metastasis, and second primary lung cancer calculated with the Kaplan-Meier method with death as a competing risk is presented in eFigure 2 in the Supplement. The cumulative rates for LR with death as a competing risk were 4% at 1 year, 9% at 3 years, and 11% at 5 years; corresponding rates for RR were 6% at 1 year, 11% at 3 years, and 12% at 5 years; for distant failure, 10% at 1 year, 18% at 3 years, and 21% at 5 years; and for second primary lung cancer, 5.9% at 1 year, 10.9% at 3 years, and 11.9% at 5 years. Rates of OS for all 912 patients were 88.8% at 1 year, 64.9% at 3 years, and 47.7% at 5 years; corresponding rates of progression-free survival were 78.1% at 1 year, 52.7% at 3 years, and 39.1% at 5 years (eFigure 3 in the Supplement).

### Characteristics of Patients Who Received Salvage Therapy for iLR or iRR

Most patients with iLR (38 of 49 [77.6%]) or iRR (40 of 53 [75.5%]) had biopsy confirmation of recurrence; 39 patients with iLR (79.6%) and 48 patients with iRR (90.6%) received salvage therapy (Table 2). Median times to recurrence after SABR were 14.5 months (range, 1.5-60.8 months) for iLR and 9.0 months (range, 1.9-70.7 months) for iRR. The median follow-up time for patients with iLR or iRR was 57.2 months (IQR, 37.7-87.6 months) from the initial SABR and 38.5 months (IQR, 19.9-69.3 months) after the recurrence. The mean time from recurrence to initiation of salvage treatment was 2.0 months (IQR, 0.0-25.4 months) for those with iLR and 1.4 months (IQR, 0.1-30.1 months) for those with iRR; this interval was used to exclude distant disease and to allow case review by the multidisciplinary team. Time to recurrence was numerically shorter for those with iRR than for those with iLR. Other characteristics between patients with iLR and those with iRR are presented in Table 2.

**Table 2.** Characteristics of All Patients With Isolated Local Recurrence (iLR) or Isolated Regional Recurrence (iRR) and Patients Without Recurrence

Characteristic	Patients, No. (%)		
	With iLR (n = 49)	With iRR (n = 53)	Without Recurrence (n = 658)
Age at recurrence, median (range), y	74 (57-89)	70 (49-89)	NA
Sex			
Male	25 (51.0)	34 (64.2)	323 (49.1)^a^
Female	24 (49.0)	19 (35.8)	335 (50.9)
ECOG score at recurrence			
0	1 (2.0)	1 (1.9)	63 (9.6)
1	35 (71.4)	36 (67.9)	481 (73.1)
2	10 (20.4)	12 (22.6)	104 (15.8)^b^
3	3 (6.1)	4 (7.5)	10 (1.5)
Tumor status (initial stage)			
T1	41 (83.7)	44 (83.0)	553 (84.0)
T2 (T2a: ≤5 cm, pleural invasion)	7 (14.3)	8 (15.1)	96 (14.6)
T3 (with satellite nodule)	1 (2.0)	1 (1.9)	9 (1.4)
Tumor histologic findings (initial)			
Adenocarcinoma	23 (46.9)	25 (47.2)	366 (55.6)
Squamous cell carcinoma	22 (44.9)	21 (39.6)	217 (33.0)
Other	1 (2.0)	2 (3.8)	14 (2.1)
NSC NOS	2 (4.1)	5 (9.4)	54 (8.2)
Unknown or no pathologic findings obtained	1 (2.0)	0	7 (1.1)
Recurrence confirmed			
Biopsy	38 (77.6)	40 (75.5)	NA
PET-CT	8 (16.3)	13 (24.5)	NA
CT	3 (6.1)	0	NA
Time to recurrence, median (range), mo	14.5 (1.5-60.8)	9.0 (1.9-70.7)	NA
Received salvage treatment, No. (%)	39 (79.6)	48 (90.6)	NA
SABR	15 (38.5)	NA	NA
Surgery	10 (25.6)	1 (2.1)	NA
Thermal ablation	6 (15.4)	NA	NA
Chemoradiotherapy	2 (5.1)	26 (54.2)	NA
Radiotherapy alone	1 (2.6)	12 (25)	NA
Systemic therapy alone	5 (12.8)	8 (16.7)	NA
Other	NA	1 (2.1)	NA
Time to salvage from time of recurrence, median (range), mo	2.0 (0.0-25.4)	1.4 (0.1-30.1)	NA

Abbreviations: CT, computed tomography; ECOG, Eastern Cooperative Oncology Group; NA, not applicable; NSC NOS, non–small cell not otherwise specified; PET-CT, positron emission tomography and computed tomography; SABR, stereotactic ablative radiotherapy.

^a^ iRR vs no recurrence, *P* = .04.

^b^ ECOG score 0-1 vs 2-3 for iRR vs no recurrence, *P* = .02.

### Salvage Therapy Characteristics and Toxic Effects

Several types of therapy were used for salvage treatment. Among patients with iLR, 15 had SABR as salvage treatment, 10 had surgery, 6 had thermal ablation, 5 had chemotherapy only, 2 had chemoradiotherapy, 1 had conventional radiotherapy, and 10 had no treatment; among patients with iRR, 26 had chemoradiotherapy, 12 had chemotherapy only, 8 had conventional radiotherapy, 1 had surgery, 1 had brachytherapy, and 5 had no treatment (eTables 1 and 2 in the Supplement). Among patients with iLR, grade 3 or greater toxic effects occurred in 1 of the 15 patients who had SABR (6.7%; pneumonitis), 4 of the 10 who had surgery (40.0%; postoperative renal, cardiac, and/or pulmonary sequelae; the 90-day mortality rate was 0% and symptoms resolved in all 4 patients), none of the 6 who had thermal ablation (0%), and 2 of the 5 patients who had systemic treatment (40.0%; hematologic). Among patients with iRR, grade 3 or greater toxic effects occurred in 10 of 26 patients who had chemoradiotherapy (38.5%; most common were esophagitis, fatigue, and hematologic effects), 1 of 8 who had conventional radiotherapy (12.5%; dyspnea), and 4 of 12 who had systemic therapy (33.3%; most common was fatigue). No patient experienced any salvage-related grade 5 event. Further details on the type and grade of toxic effects experienced for each salvage treatment can be found in eTables 1 and 2 in the Supplement.

Although systemic therapy alone was not considered definitive for local salvage, it was included as a form of salvage treatment in this study given its presumed role in reducing morbidity and mortality from recurrent disease. One patient with iRR had brachytherapy as salvage treatment for mediastinal recurrence invading the trachea, and another underwent surgery for a low disease burden. Two patients with iLR received chemoradiotherapy, one for aggressive management and the other as induction therapy to reduce the size of the radiotherapy field.

### Survival After iLR and iRR

**Figure 2.**
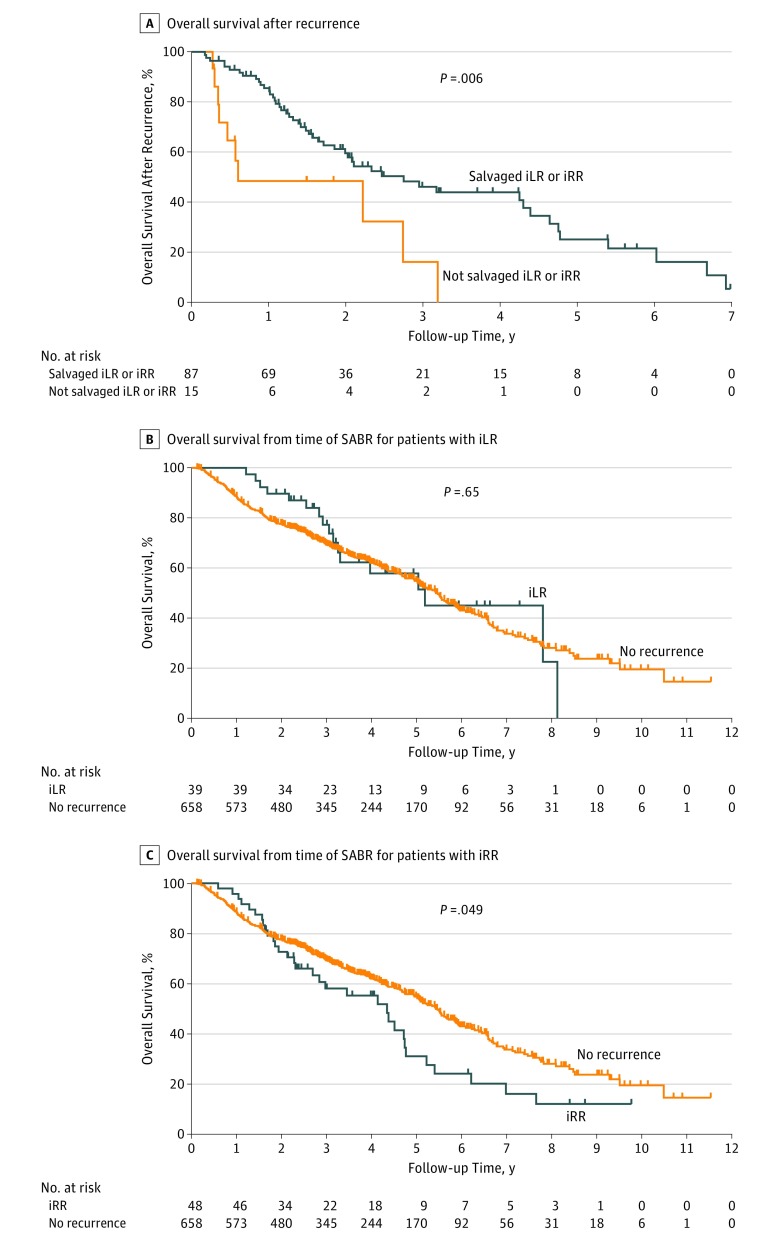
Survival Outcomes After Salvage Therapy for Isolated Local Recurrence (iLR) or Isolated Regional Recurrence (iRR) A, Overall survival after recurrence for patients with iLR or iRR who did or did not undergo salvage therapy. B, Overall survival from the time of initial stereotactic ablative radiation therapy (SABR) for patients with iLR who received salvage treatment vs for patients with no recurrence. C, Overall survival from the time of initial SABR for patients with iRR who received salvage treatment vs for patients with no recurrence.

Overall survival time was significantly longer for patients with iLR or iRR who received salvage treatment (n = 87) than for those with iLR or iRR who did not receive salvage treatment (n = 15) (37 vs 7 months from time of recurrence; hazard ratio [HR], 0.40; 95% CI, 0.09-0.66; *P* = .006; Figure 2A). Rates of OS after recurrence for patients with iLR plus salvage treatment were 92.0% at 1 year, 55.3% at 3 years, and 33.2% at 5 years (Figure 2B); for patients with iRR plus salvage treatment, rates of OS were 80.3% at 1 year, 40.4% at 3 years, and 20.7% at 5 years (Figure 2C). Rates of OS after recurrence for patients with untreated iLR were lower, at 64.8% at 1 year, 34.2% at 3 years, and 0% at 5 years; for patients with untreated iRR, rates of OS were 20.0% at 1 year, 0% at 3 years, and 0% at 5 years (Figure 2A).

When evaluating whether salvageable recurrence adversely affects survival, we found that OS was no different for patients with iLR who received salvage treatment than for patients who had no recurrence after initial SABR (log-rank *P* = .65); rates of OS at 5 years from initial SABR were no different between patients with iLR and salvage treatment (57.9%) and patients with no recurrence (54.9%; HR, 0.89; 95% CI, 0.56-1.43; time-varying *P* = .10; HR, 1.51; 95% CI, 0.92-2.47; Figure 2B and Table 3). However, rates of OS at 5 years for patients with iRR who received salvage treatment (31.1%) were significantly lower than those for patients with no recurrence (log-rank *P* = .049; HR, 1.43; 95% CI, 1.00-2.34; time-varying *P* < .001; HR, 2.08; 95% CI, 1.45-3.01; Figure 2C and Table 3).

**Table 3.** Outcomes of Patients After Salvage for Isolated Local Recurrence (iLR) or Isolated Regional Recurrence (iRR) Compared With Patients Without Recurrence

Characteristic	Patients With iLR (n = 39)	Patients With iRR (n = 48)	Patients Without Recurrence (n = 658)
OS time from initial SABR, median (95% CI), mo	62.3 (38.2-86.4)	52.3 (35.3-69.2)	65.3 (60.3-70.3)^a^^,^^b^
OS rate, %			
1 y	97.4	95.8	88.8
3 y	77.3	58.1	69.5
5 y	57.9	31.1	54.9
OS time after recurrence, median (95% CI), mo	51.6 (0.0-110.6)	28.0 (14.6-41.5)	
OS rate, %			
1 y	92.0	80.3	NA
3 y	55.3	40.4	NA
5 y	33.2	20.7	NA
Cumulative subsequent recurrence for patients after salvage, No. (%)			
LR	7 (17.9)	1 (2.1)	NA
RR	9 (23.1)	2 (4.2)	NA
DM	10 (25.6)	14 (29.2)	NA
Time to subsequent event for patients after salvage, median (range), mo			
LR	11.8 (2.7-20.8)	9.6^c^	NA
RR	10.3 (1.2-16.2)	23.4 (12.1-34.6)	NA
DM	12.5 (0.3-78.6)	8.3 (1.2-34.6)	NA
Location of distant metastases, No./total No. (%)			
Intrathoracic	9/10 (90.0)	8/14 (57.1)	NA
Extrathoracic	1/10 (10.0)	6/14 (42.9)	NA

Abbreviations: DM, distant mestastasis; LR local recurrence; NA, not applicable; OS, overall survival; RR, regional recurrence; SABR, stereotactic ablative radiotherapy.

^a^ For iLR vs no recurrence, *P* = .65 by Kaplan-Meier analysis, and *P* = .10 by time-varying analysis.

^b^ For iRR vs no recurrence, *P* = .049 by Kaplan-Meier analysis, and *P* < .001 by time-varying analysis.

^c^ There is no range because there was only 1 patient.

### Disease Progression After Salvage Therapy

Subsequent recurrence events after salvage are presented in Table 3. Nineteen of 39 patients with iLR (48.7%) and 33 of 48 patients with iRR (68.8%) had no further recurrence. Subsequent LR occurred in 7 patients with iLR (17.9%) and 1 patient with iRR (2.1%), subsequent RR occurred in 9 patients with iLR (23.1%) and in 2 patients with iRR (4.2%), and subsequent distant failure occurred in 10 patients with iLR (25.6%) and 14 patients with iRR (28.6%). Sites of distant failure differed for patients with iLR (9 of 10 [90.0%], lungs and 1 of 10 [10.0%], extrathoracic) vs iRR (8 of 14 [57.1%], lungs and 6 of 14 [42.9%], extrathoracic): extrathoracic sites included the bone, liver, adrenal glands, and brain. All patients with iLR or iRR who did not receive salvage therapy had progressive disease (n = 15).

## Discussion

Our key findings from this large study of long-term outcomes after salvage treatment for locally or regionally recurrent disease after SABR for early-stage NSCLC are as follows. First, life expectancy for patients with iLR after SABR who subsequently received salvage treatment was virtually the same as that for patients without recurrence. Moreover, at 3 years after recurrence, 50% to 60% of patients with iLR or iRR who received salvage treatment never had another recurrence, showing that the potential cure rate with salvage treatment for such patients can be substantial. The OS for patients with iRR was poorer than that for patients with iLR or no recurrence, but was akin to that for patients with stage III disease. Thus, although salvage treatment offers better outcomes as a whole, iLR and iRR represent 2 distinct clinical paths, an important distinction for clinicians managing such cases.

We further found support for using salvage treatment, because patients receiving any salvage had better OS than patients who did not. Although one might expect survival in patients who did not receive salvage treatment to be poorer (perhaps because comorbidities precluded salvage), we found that all patients with recurrence who did not receive salvage treatment experienced progression and none were alive at 3 to 5 years after recurrence.

For those who experienced progression after salvage treatment, that progression was mostly distant, and sites varied between the 2 recurrence groups. Specifically, 90% of recurrences after salvage treatment for iLR occurred in a different lung lobe, whereas distant failure for patients with iRR who received salvage treatment was more often extrathoracic and disseminated, which may have contributed to the poorer OS in the iRR subgroup.

Patients with iLR who received salvage treatment had higher rates of subsequent LR and RR events than did patients with iRR who received salvage treatment. This result was not surprising because local lobar disease was apparently controlled in most patients with iRR but not in patients with iLR. Furthermore, unlike patients with iRR, most patients with iLR did not receive nodal or mediastinal sterilizing therapy (ie, chemoradiotherapy), which could make regional nodes the most logical location for recurrence, should recurrence take place. Subsequent LR and RR was managed with the same approach as that for initial salvage treatment, likely contributing to the favorable OS for patients with iLR.

Although most patients achieved disease control, the 40% rate of recurrence after salvage treatment suggests the potential for systemic therapy upfront for patients with either iLR or iRR to eradicate distant or residual microscopic disease at the time of recurrence. To this end, the addition of immunotherapy to SABR for patients with newly diagnosed early-stage disease or iLR after SABR (I-SABR [ClinicalTrials.gov identifier NCT03110978]) is being tested.^38^ Finally, we showed that a variety of salvage techniques, including thermal ablation (not currently included in national guidelines), could be successful for patients who are unable to undergo other locally directed therapies.

### Limitations

This retrospective review provides data on outcomes for patients with recurrence after SABR. Our approach is similar to the National Comprehensive Cancer Network consensus algorithm. Our study has some limitations, chief among them its retrospective nature, with all the inherent biases. Any nonrandomized comparison of the effectiveness of various salvage techniques is limited by bias in assigning patients to a particular salvage therapy based on performance and disease status. Our results, based on a low rate (11%) of iLR or iRR in patients undergoing SABR, indicate that a prospective clinical trial would be a challenge. Finally, the single-institution nature of this study was both a limitation and a strength in that it allowed a relatively complete long-term analysis.

## Conclusions

Life expectancy after salvage treatment for iLR was similar to that for patients without recurrence, but survival after salvage treatment for iRR was similar to that of patients with stage III NSCLC. Because salvage treatment for iLR or iRR was based on a consistent multidisciplinary approach, the results of this study may help clinicians and patients in treatment decision making.

## Notes

Supplement. **eFigure 1.** CONSORT-Style Diagram Showing Patterns of First Recurrence **eFigure 2.** Cumulative Incidence of Local Recurrence (LR), Regional Recurrence (RR), Distant Metastasis (DM), and Second Primary Lung Cancer (SPLC) After Definitive Stereotactic Ablative Radiation Therapy (SABR) Among 912 Patients With Early-stage Non-Small Cell Lung Cancer **eFigure 3.** Progression-Free Survival (PFS) (A) and Overall Survival (B) Outcomes for 912 Patients, Beginning at the Completion of Stereotactic Ablative Radiation Therapy for Early-stage Non-Small Cell Lung Cancer **eTable 1.** Salvage Therapy for Isolated Local Recurrences **eTable 2.** Salvage Therapy for Isolated Regional Recurrence

## References

[1] Timmerman R, Paulus R, Galvin J, Stereotactic body radiation therapy for inoperable early stage lung cancer. JAMA. 2010;303(11):-. doi:10.1001/jama.2010.261 20233825 PMC2907644

[2] Chang JY, Senan S, Paul MA, Stereotactic ablative radiotherapy versus lobectomy for operable stage I non–small-cell lung cancer: a pooled analysis of two randomised trials. Lancet Oncol. 2015;16(6):630-637. doi:10.1016/S1470-2045(15)70168-3 25981812 PMC4489408

[3] Sun B, Brooks ED, Komaki RU, 7-Year follow-up after stereotactic ablative radiotherapy for patients with stage I non-small cell lung cancer: results of a phase 2 clinical trial. Cancer. 2017;123(16):3031-3039. doi:10.1002/cncr.30693 28346656 PMC5544582

[4] Senthi S, Lagerwaard FJ, Haasbeek CJ, Slotman BJ, Senan S Patterns of disease recurrence after stereotactic ablative radiotherapy for early stage non–small-cell lung cancer: a retrospective analysis. Lancet Oncol. 2012;13(8):802-809. doi:10.1016/S1470-2045(12)70242-5 22727222

[5] Smith BD, Smith GL, Hurria A, Hortobagyi GN, Buchholz TA Future of cancer incidence in the United States: burdens upon an aging, changing nation. J Clin Oncol. 2009;27(17):2758-2765. doi:10.1200/JCO.2008.20.8983 19403886

[6] Holmes JA, Zagar TM, Chen RC Adoption of stereotactic body radiotherapy for stage IA non-small cell lung cancer across the United States. J Natl Cancer Inst Cancer Spectrum. 2017;1(1):pkx003. 10.1093/jncics/pkx003 PMC6649706 31360829

[7] National Comprehensive Cancer Network Non-small cell lung cancer (version 2.2018). https://www.nccn.org/professionals/physician_gls/pdf/nscl.pdf. Accessed February 2, 2018.

[8] European Society for Medical Oncology ESMO clinical practice guidelines: lung and chest tumors. http://www.esmo.org/Guidelines/Lung-and-Chest-Tumours. Accessed February 2, 2018.

[9] Verstegen NE, Lagerwaard FJ, Hashemi SM, Dahele M, Slotman BJ, Senan S Patterns of disease recurrence after SABR for early stage non–small-cell lung cancer: optimizing follow-up schedules for salvage therapy. J Thorac Oncol. 2015;10(8):1195-1200. doi:10.1097/JTO.0000000000000576 26200274

[10] Verstegen NE, Maat AP, Lagerwaard FJ, Salvage surgery for local failures after stereotactic ablative radiotherapy for early stage non-small cell lung cancer. Radiat Oncol. 2016;11(1):131. doi:10.1186/s13014-016-0706-7 27716240 PMC5048455

[11] McAvoy S, Ciura K, Wei C, Definitive reirradiation for locoregionally recurrent non-small cell lung cancer with proton beam therapy or intensity modulated radiation therapy: predictors of high-grade toxicity and survival outcomes. Int J Radiat Oncol Biol Phys. 2014;90(4):819-827. doi:10.1016/j.ijrobp.2014.07.030 25220718

[12] Hearn JW, Videtic GM, Djemil T, Stephans KL Salvage stereotactic body radiation therapy (SBRT) for local failure after primary lung SBRT. Int J Radiat Oncol Biol Phys. 2014;90(2):402-406. doi:10.1016/j.ijrobp.2014.05.048 25017480

[13] Trakul N, Harris JP, Le QT, Stereotactic ablative radiotherapy for reirradiation of locally recurrent lung tumors. J Thorac Oncol. 2012;7(9):1462-1465. doi:10.1097/JTO.0b013e31825f22ce 22895143

[14] Kilburn JM, Kuremsky JG, Blackstock AW, Thoracic re-irradiation using stereotactic body radiotherapy (SBRT) techniques as first or second course of treatment. Radiother Oncol. 2014;110(3):505-510. doi:10.1016/j.radonc.2013.11.017 24444530 PMC4006197

[15] Kelly P, Balter PA, Rebueno N, Stereotactic body radiation therapy for patients with lung cancer previously treated with thoracic radiation. Int J Radiat Oncol Biol Phys. 2010;78(5):1387-1393. doi:10.1016/j.ijrobp.2009.09.070 20381271 PMC3401019

[16] Meijneke TR, Petit SF, Wentzler D, Hoogeman M, Nuyttens JJ Reirradiation and stereotactic radiotherapy for tumors in the lung: dose summation and toxicity. Radiother Oncol. 2013;107(3):423-427. doi:10.1016/j.radonc.2013.03.015 23647748

[17] Peulen H, Karlsson K, Lindberg K, Toxicity after reirradiation of pulmonary tumours with stereotactic body radiotherapy. Radiother Oncol. 2011;101(2):260-266. doi:10.1016/j.radonc.2011.09.012 22056534

[18] Reyngold M, Wu AJ, McLane A, Toxicity and outcomes of thoracic re-irradiation using stereotactic body radiation therapy (SBRT). Radiat Oncol. 2013;8(1):99. doi:10.1186/1748-717X-8-99 23617949 PMC3651392

[19] Bauman JE, Mulligan MS, Martins RG, Kurland BF, Eaton KD, Wood DE Salvage lung resection after definitive radiation (>59 Gy) for non-small cell lung cancer: surgical and oncologic outcomes. Ann Thorac Surg. 2008;86(5):1632-1638. doi:10.1016/j.athoracsur.2008.07.042 19049763

[20] Allibhai Z, Cho BC, Taremi M, Surgical salvage following stereotactic body radiotherapy for early-stage NSCLC. Eur Respir J. 2012;39(4):1039-1042. doi:10.1183/09031936.00075811 22467727

[21] Chen F, Matsuo Y, Yoshizawa A, Salvage lung resection for non-small cell lung cancer after stereotactic body radiotherapy in initially operable patients. J Thorac Oncol. 2010;5(12):1999-2002. doi:10.1097/JTO.0b013e3181f260f9 21102261

[22] Antonoff MB, Correa AM, Sepesi B, Salvage pulmonary resection after stereotactic body radiotherapy: a feasible and safe option for local failure in selected patients. J Thorac Cardiovasc Surg. 2017;154(2):689-699. doi:10.1016/j.jtcvs.2017.03.142 28495066

[23] Sun B, Brooks ED, Komaki R, Long-term outcomes of salvage stereotactic ablative radiotherapy for isolated lung recurrence of non-small cell lung cancer: a phase II clinical trial. J Thorac Oncol. 2017;12(6):983-992. doi:10.1016/j.jtho.2017.02.018 28259750 PMC5881570

[24] von Elm E, Altman DG, Egger M, Pocock SJ, Gøtzsche PC, Vandenbroucke JP; STROBE Initiative The Strengthening the Reporting of Observational Studies in Epidemiology (STROBE) statement: guidelines for reporting observational studies. J Clin Epidemiol. 2008;61(4):344-349. doi:10.1016/j.jclinepi.2007.11.008 18313558

[25] Chang JY, Li QQ, Xu QY, Stereotactic ablative radiation therapy for centrally located early stage or isolated parenchymal recurrences of non-small cell lung cancer: how to fly in a ‘no fly zone’. Int J Radiat Oncol Biol Phys. 2014;88(5):1120-1128. doi:10.1016/j.ijrobp.2014.01.022 24661665

[26] Zhang X, Liu H, Balter P, Positron emission tomography for assessing local failure after stereotactic body radiotherapy for non–small-cell lung cancer. Int J Radiat Oncol Biol Phys. 2012;83(5):1558-1565. doi:10.1016/j.ijrobp.2011.10.035 22572078 PMC3474601

[27] Zhao L, Zhou S, Balter P, Planning target volume D95 and mean dose should be considered for optimal local control for stereotactic ablative radiation therapy. Int J Radiat Oncol Biol Phys. 2016;95(4):1226-1235. doi:10.1016/j.ijrobp.2016.01.065 27209498

[28] Li Q, Swanick CW, Allen PK, Stereotactic ablative radiotherapy (SABR) using 70 Gy in 10 fractions for non-small cell lung cancer: exploration of clinical indications. Radiother Oncol. 2014;112(2):256-261. doi:10.1016/j.radonc.2014.07.010 25108807

[29] Martini N, Melamed MR Multiple primary lung cancers. J Thorac Cardiovasc Surg. 1975;70(4):606-612. 170482

[30] Dupuy DE, Zagoria RJ, Akerley W, Mayo-Smith WW, Kavanagh PV, Safran H Percutaneous radiofrequency ablation of malignancies in the lung. AJR Am J Roentgenol. 2000;174(1):57-59. doi:10.2214/ajr.174.1.1740057 10628454

[31] Fernando HC, De Hoyos A, Landreneau RJ, Radiofrequency ablation for the treatment of non-small cell lung cancer in marginal surgical candidates. J Thorac Cardiovasc Surg. 2005;129(3):639-644. doi:10.1016/j.jtcvs.2004.10.019 15746749

[32] de Baère T, Palussière J, Aupérin A, Midterm local efficacy and survival after radiofrequency ablation of lung tumors with minimum follow-up of 1 year: prospective evaluation. Radiology. 2006;240(2):587-596. doi:10.1148/radiol.2402050807 16864679

[33] Lee JM, Jin GY, Goldberg SN, Percutaneous radiofrequency ablation for inoperable non-small cell lung cancer and metastases: preliminary report. Radiology. 2004;230(1):125-134. doi:10.1148/radiol.2301020934 14645875

[34] Temel JS, Greer JA, Muzikansky A, Early palliative care for patients with metastatic non–small-cell lung cancer. N Engl J Med. 2010;363(8):733-742. doi:10.1056/NEJMoa1000678 20818875

[35] Verduijn M, Grootendorst DC, Dekker FW, Jager KJ, le Cessie S The analysis of competing events like cause-specific mortality—beware of the Kaplan-Meier method. Nephrol Dial Transplant. 2011;26(1):56-61. doi:10.1093/ndt/gfq661 21059831

[36] US Dept of Health and Human Services Common terminology criteria for adverse events (CTCAE): version 4.0. https://evs.nci.nih.gov/ftp1/CTCAE/CTCAE_4.03/CTCAE_4.03_2010-06-14_QuickReference_5x7.pdf. Published May 28, 2009. Accessed July 2, 2018.

[37] Edge SB, Byrd DR, Compton CC, Fritz AG, Greene FL, Trotti A, eds. AJCC Cancer Staging Manual. 7th ed New York, NY: Springer; 2010.

[38] Bernstein MB, Krishnan S, Hodge JW, Chang JY Immunotherapy and stereotactic ablative radiotherapy (ISABR): a curative approach? Nat Rev Clin Oncol. 2016;13(8):516-524. doi:10.1038/nrclinonc.2016.30 26951040 PMC6053911

