# Supplementary material for: Association of Long-term Outcomes and Survival With Multidisciplinary Salvage Treatment for Local and Regional Recurrence After Stereotactic Ablative Radiotherapy for Early-Stage Lung Cancer
Source: JAMA Netw Open. 2018 Aug 24;1(4):e181390. doi: 10.1001/jamanetworkopen.2018.1390 (PMC6324276; doi:10.1001/jamanetworkopen.2018.1390)
Supplement: Supplement. — eFigure 1. CONSORT-Style Diagram Showing Patterns of First Recurrence eFigure 2. Cumulative Incidence of Local Recurrence (LR), Regional Recurrence (RR), Distant Metastasis (DM), and Second Primary Lung Cancer (SPLC) After Definitive Stereotactic Ablative Radiation Therapy (SABR) Among 912 Patients With Early-stage Non-Small Cell Lung Cancer eFigure 3. Progression-Free Survival (PFS) (A) and Overall Survival (B) Outcomes for 912 Patients, Beginning at the Completion of Stereotactic Ablative Radiation Therapy for Early-stage Non-Small Cell Lung Cancer eTable 1. Salvage Therapy for Isolated Local Recurrences eTable 2. Salvage Therapy for Isolated Regional Recurrence [file jamanetwopen-1-e181390-s001.pdf]

Supplementary Online Content

Brooks ED, Sun B, Feng L, et al. Association of long-term outcomes and survival with multidisciplinary salvage treatment for local and regional recurrence after stereotactic ablative radiotherapy for early-stage lung cancer. *JAMA Netw Open*. 2018;1(4):e181390. doi:10.1001/jamanetworkopen.2018.1390

eFigure 1. CONSORT-Style Diagram Showing Patterns of First Recurrence
eFigure 2. Cumulative Incidence of Local Recurrence (LR), Regional Recurrence (RR), Distant Metastasis (DM), and Second Primary Lung Cancer (SPLC) After Definitive Stereotactic Ablative Radiation Therapy (SABR) Among 912 Patients With Early-stage Non-Small Cell Lung Cancer
eFigure 3. Progression-Free Survival (PFS) (A) and Overall Survival (B) Outcomes for 912 Patients, Beginning at the Completion of Stereotactic Ablative Radiation Therapy for Early-stage Non-Small Cell Lung Cancer
eTable 1. Salvage Therapy for Isolated Local Recurrences
eTable 2. Salvage Therapy for Isolated Regional Recurrence

This supplementary material has been provided by the authors to give readers additional information about their work.

© 2018 Brooks ED et al. *JAMA Network Open.*

eFigure 1. CONSORT-Style Diagram Showing Patterns of First Recurrence

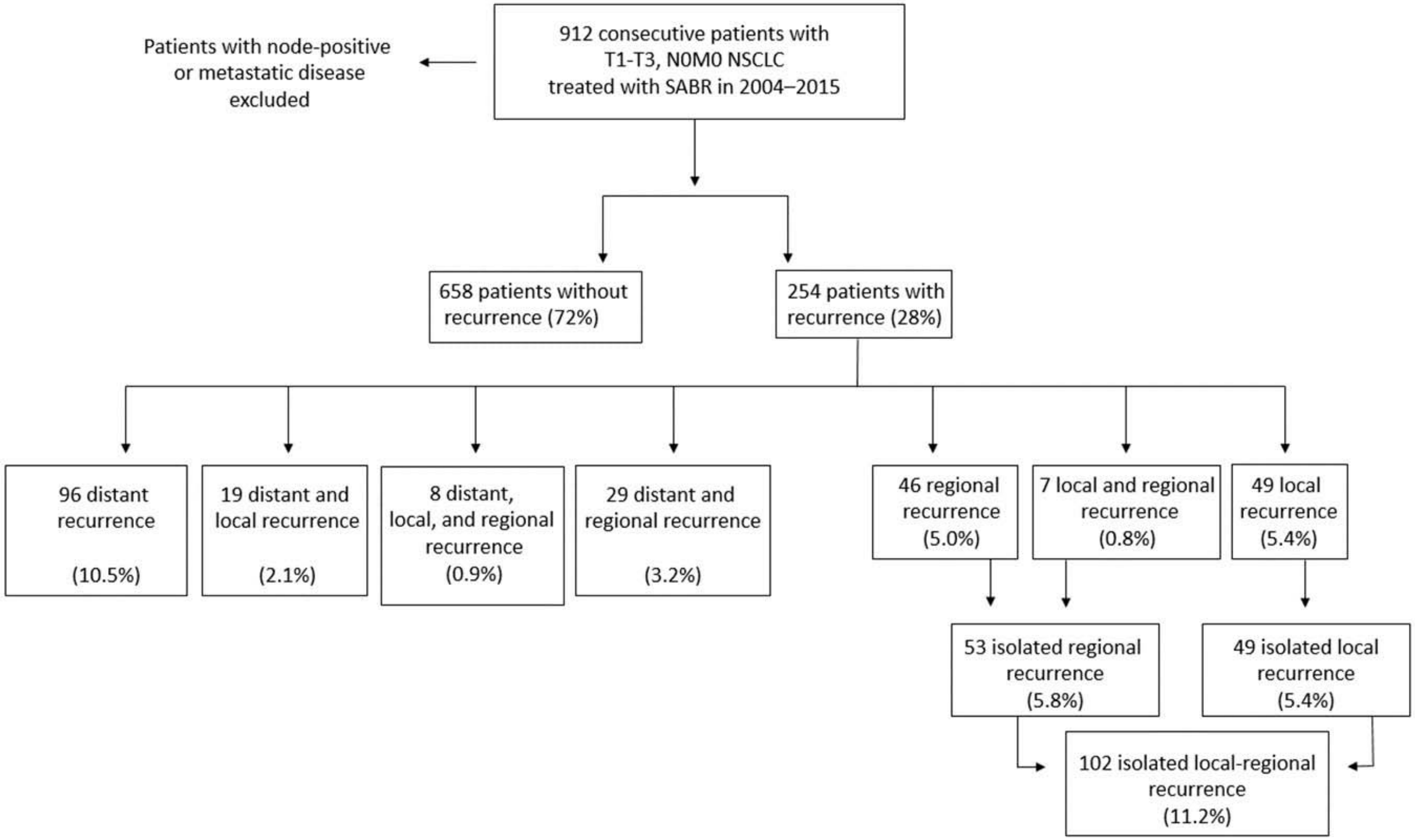

Values shown are numbers of patients with local, regional, or distant recurrence, followed by percentages of patients experiencing each type of recurrence.

© 2018 Brooks ED et al. *JAMA Network Open.*

eFigure 2. Cumulative Incidence of Local Recurrence (LR), Regional Recurrence (RR), Distant Metastasis (DM), and Second Primary Lung Cancer (SPLC) After Definitive Stereotactic Ablative Radiation Therapy (SABR) Among 912 Patients With Early-stage Non-Small Cell Lung Cancer

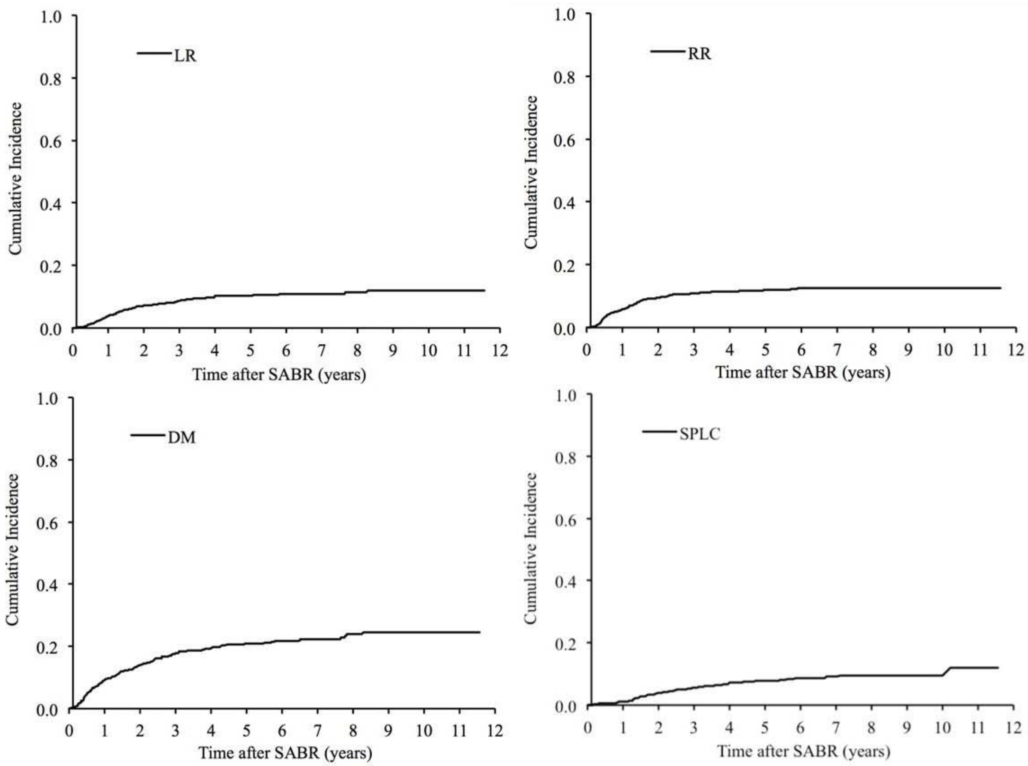

The Kaplan-Meier method was used, with death as a competing risk

© 2018 Brooks ED et al. *JAMA Network Open.*

eFigure 3. Progression-Free Survival (PFS) (A) and Overall Survival (B) Outcomes for 912 Patients, Beginning at the Completion of Stereotactic Ablative Radiation Therapy for Early-stage Non-Small Cell Lung Cancer

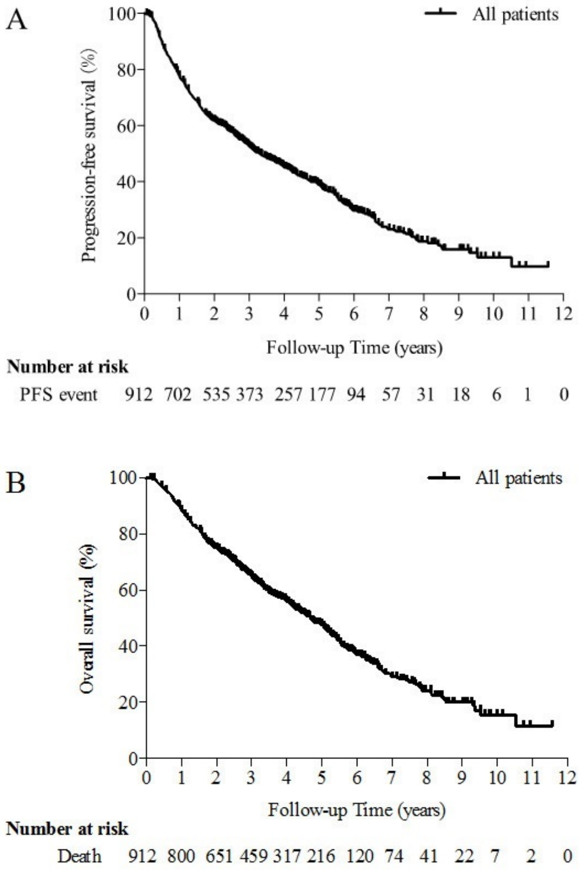

© 2018 Brooks ED et al. *JAMA Network Open.*

eTable 1. Salvage Therapy for Isolated Local Recurrences (iLR)

**Treatment Characteristics**	**SABR** (n=15)	**Surgery** (n=10)	**Thermal Ablation** (n=6)	**Chemotherapy Only** (n=5)	**Chemo-radiation** (n=2)	**Conventional Radiation** (n=1)	**No Treatment** (n=10)
**Types of treatment**, no.	50 Gy in 4 fx, 9	Lobectomy, 6	Percutaneous RFA	Platinum+ paclitaxel, 1	Platinum+ paclitaxel, 2	45 Gy in 15 fx, 1	—
	60 Gy in 10-15 fx, 3	Sublobar, 4		IL-10, 1	70 Gy in 35 fx, 1		
	70 Gy in 10 fx, 3			Erlotinib, 3	45 Gy in 15 fx, 1		
**Reasons for treatment**	Preferred treatment, 100%	Not a candidate for re-irradiation, 100%	Not a candidate for surgery or re-irradiation, 100%	Not a candidate for surgery, re-irradiation, or local therapy, 100%	Opted for aggressive chemoRT, 50%	Recurrence in parenchyma close to hilum, not candidate for SABR or surgery, 100%	Declined treatment, 40%
					Induction to enable safe re-irradiation, 50%		Not a candidate for further treatment, 60%
**Recurrence type**	11 out-of-field	6 in-field	6 in-field				
	4 marginal	4 marginal					
**Local and regional control rates for definitive therapy based on first events**	LC: 87%	LC: 100%	LC: 100%	--	--	--	--
	RC: 80%	RC: 90%	RC: 83.4%				
**Patients with severe (grade**	6.6%	40%*	—	40%	50%	—	—

© 2018 Brooks ED et al. *JAMA Network Open.*

**≥3) toxicity**, (%)†							
**Treatment Characteristics**	**SABR** (n=15)	**Surgery** (n=10)	**Thermal Ablation** (n=6)	**Chemotherapy Only** (n=5)	**Chemo-radiation** (n=2)	**Conventional Radiation** (n=1)	**No Treatment** (n=10)
**Toxicity type**, no.	Pneumonitis, 1	Fatigue, 1	—	Platinum+ Paclitaxel	Dose reduction, 1	—	—
		Respiratory Distress, 1		Hospitalization, 1	Fatigue, 1		
		Anemia, 1		Anemia, 1			
		Renal insufficiency, 1		Thrombo-cytopenia, 1			
		Pleural effusion, 1		Erlotinib			
		Atrial fibrillation, 1		—			
		Ileus/enterocolitis, 1		IL-10			
				Pneumonitis, 1			
				Hospitalization, 1			

Abbreviations: SABR, stereotactic ablative radiotherapy; fx, fractions; RFA, radiofrequency ablation; LC, local control; RC, regional control

*The 90-day mortality rate was 0%; all patients recovered from morbidity

†No patient had a grade 5 event, and all adverse events were successfully managed medically

© 2018 Brooks ED et al. *JAMA Network Open.*

eTable 2. Salvage Therapy for Isolated Regional Recurrence (iRR)

	**Chemoradiation** (n=26)	**Chemotherapy Only** (n=12)	**Conventional 3D Radiation** (n=8)	**Surgery** (n=1)	**Brachytherapy** (n=1)	**No Treatment** (n=5)
**Treatment types**, no.	Platinum+ paclitaxel, 21	Pembrolizumab, 1	70 Gy in 35 fx, 2	Lobectomy, 1	30 Gy in 2 fx (15 Gy each)	—
	Platinum+ pemetrexed, 2	Platinum+ paclitaxel, 4	60 Gy in 30 fx, 1			
	Platinum only, 2	Platinum+ gemcitabine, 1	60 Gy in 15 fx, 1			
	Paclitaxel only, 1	Erlotinib, 3	52.50 Gy in 15 fx, 2			
	70 Gy in 35 fx, 8	Platinum+ etoposide, 1	45 Gy in 15 fx, 2			
	60-66 Gy in 30-33 fx, 16	Pemetrexed, 1				
	60 Gy in 15 fx, 1					
	52.5 Gy in 15 fx, 1					
**Reasons for Treatment, n (%)**	Preferred treatment, 26 (100)	Could not tolerate re-irradiation, 11 (91)	Could not tolerate chemotherapy, 5 (66)	Not a candidate for re-irradiation; had both LR and RR (100)	Involvement and erosion of mediastinal tumor into the trachea, (100)	Declined treatment or deferred, 2 (40)
		Preferred chemo only, 1 (9%)	Declined or deferred chemotherapy, 3 (33%)			Not a candidate for further treatment, 3 (60%)
**Local and regional control rates for definitive therapy based on first events**	LC: 96.2%	--	LC: 100%	LC: 100%	--	--
	RC: 92.3%		RC: 100%	RC: 100%		
	**Chemoradiation**	**Chemotherapy**	**Conventional 3D**	**Surgery**	**Brachytherapy**	**No Treatment**

© 2018 Brooks ED et al. *JAMA Network Open.*

	(n=26)	**Only** (n=12)	**Radiation** (n=8)	(n=1)	(n=1)	(n=5)
**Patients with severe (grade ≥3) toxicity**‡	38.4%	33.3%	12.5%	100%*	0%	—
**Toxicity type**, no.	Fatigue, 4	Doublet chemo	Dyspnea, 1	Pneumonia, 1	—	—
	Hospitalization, 7	Anemia, 2				
	Premature cessation of chemo, 2	Delayed/Break in chemo, 3				
	Anorexia, 1	Fatigue, 3				
	Delay/Break in chemo, 7	Dose reduction, 1				
	Dose reduction, 1	Erlotinib				
	Rash, 1	—				
	Esophagitis, 3	Pembrolizumab				
	Pneumonitis, 1	—				
	Neutropenia, 3	Pemetrexed				
	Thrombocytopenia, 1	—				

Abbreviations: SABR, stereotactic ablative radiotherapy; fx, fractions; LC, local control; RC, regional control

*90-day mortality rate was 0%; the patient fully recovered from postoperative pneumonia

‡No patients had grade 5 events, and all adverse events were successfully managed medically

Life-threatening rash due to amifostine

© 2018 Brooks ED et al. *JAMA Network Open.*
